# Supplementary material for: The plant pathogen Pectobacterium atrosepticum contains a functional formate hydrogenlyase‐2 complex
Source: Mol Microbiol. 2019 Sep 10;112(5):1440–52. doi: 10.1111/mmi.14370 (PMC7384014; doi:10.1111/mmi.14370)
Supplement: Supplementary file 1 [file MMI-112-1440-s001.docx]

**The plant pathogen *Pectobacterium atrosepticum* contains a functional formate hydrogenlyase-2 complex.**

Alexander J. Finney, Rebecca Lowden, Michal Fleszar, Marta Albareda, Sarah J. Coulthurst, Frank Sargent

**SUPPLEMENTARY INFORMATION**


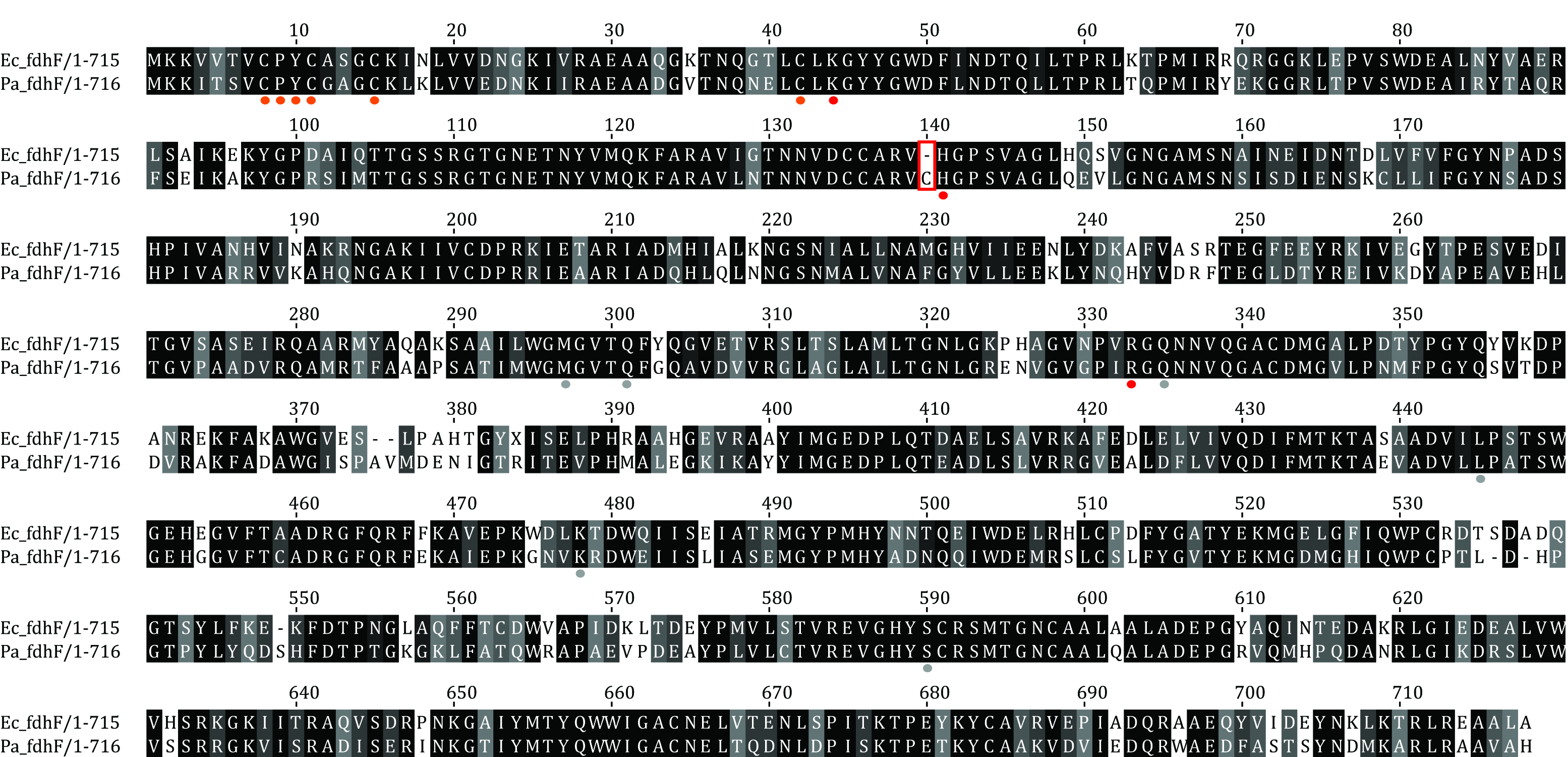


**Supp Figure S1: A selenocysteine-free formate dehydrogenase in *P. atrosepticum***

Primary sequence alignment of *E. coli* FdhF and *P. atrosepticum* SCRI1043 FdhF (ECA1250) using Jalview. Orange annotations show Fe-S cluster co-ordination residues, Red annotations show active site residues and Grey annotations show Mo-*bis*MGD co-ordination – all based on the *E. coli* FdhF crystal structure (1AA6). A red box highlights the selenocysteine/cysteine key difference between the two homologs.

**
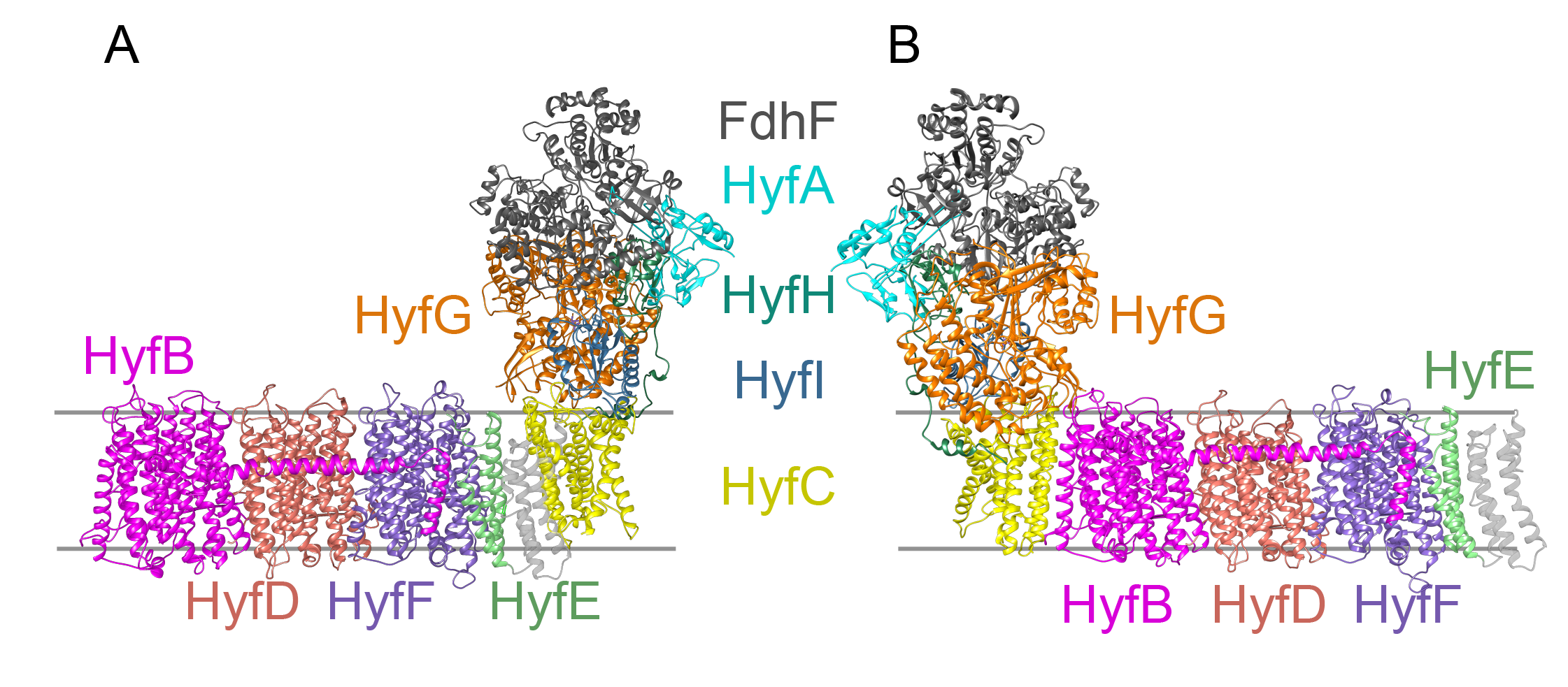
**

**Supp Figure S2: Predicted structures of formate hydrogenlyase-2.**

Structural models of formate hydrogenlyase-2 (FHL-2) from *P. atrosepticum* with **(A)** a membrane domain (membrane arm) with proteins in the order observed for Complex I, i.e. the HyfC protein is separated from HyfB by the extra membrane proteins. **(B)** Formate hydrogenlyase-2 (FHL-2) from *P. atrosepticum* with the membrane domain (membrane arm) in an architecture to the Hyd-4-like enzyme from *P. furiosus* (MBH). In this case, HyfB remains in contact with HydC and the extra membrane proteins extend away.


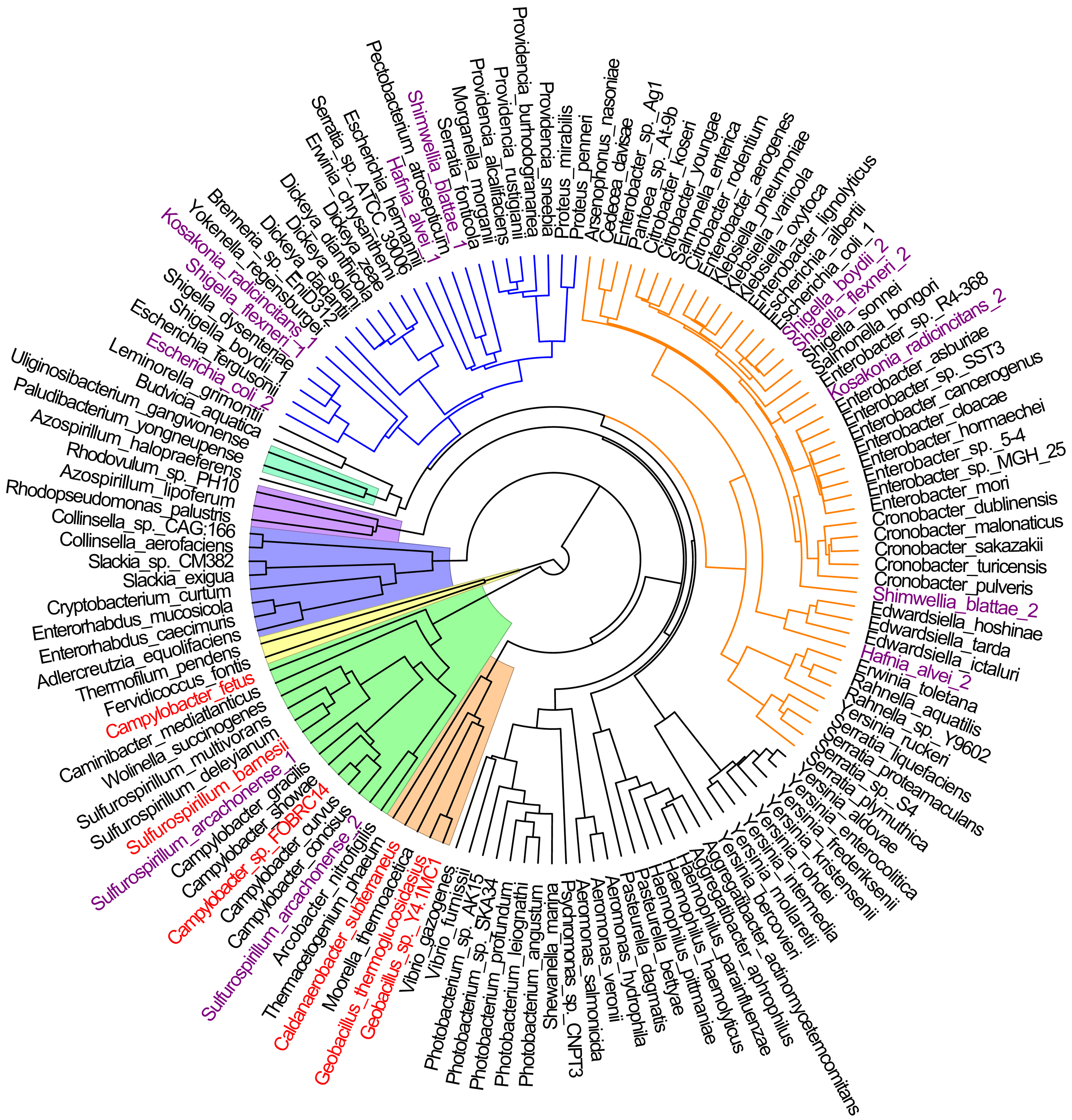


**Supp Figure S3: Phylogenetic analysis of Group 4A [NiFe]-hydrogenase catalytic subunits**

A phylogenetic tree of Group4A [NiFe]-hydrogenase catalytic subunits. Proteins linked by the orange line are those predicted to be of the HycE-type (Hyd-3), while those linked by the blue line are those predicted to be of the HyfG type (Hyd-4). The coloured sections represent different phylogenetic groups: brown section are Firmicutes (five members represented); green section are the Epsilonproteobacteria (14); yellow section are the Crenarchaeota (Archaea) (two); lilac section are Actinobacteria (eight); purple section are Alphaproteobacteria (three + *Azospirillum halopraeferens*); light blue section are Betaproteobacteria (two); and those with no highlight colour are the Gammaproteobacteria. Species names highlighted in purple text have genomes where no FdhF (formate dehydrogenase) more than one Group4A hydrogenase was identified, and species names highlighted in red text have genomes where no FdhF (formate dehydrogenase) was identified.


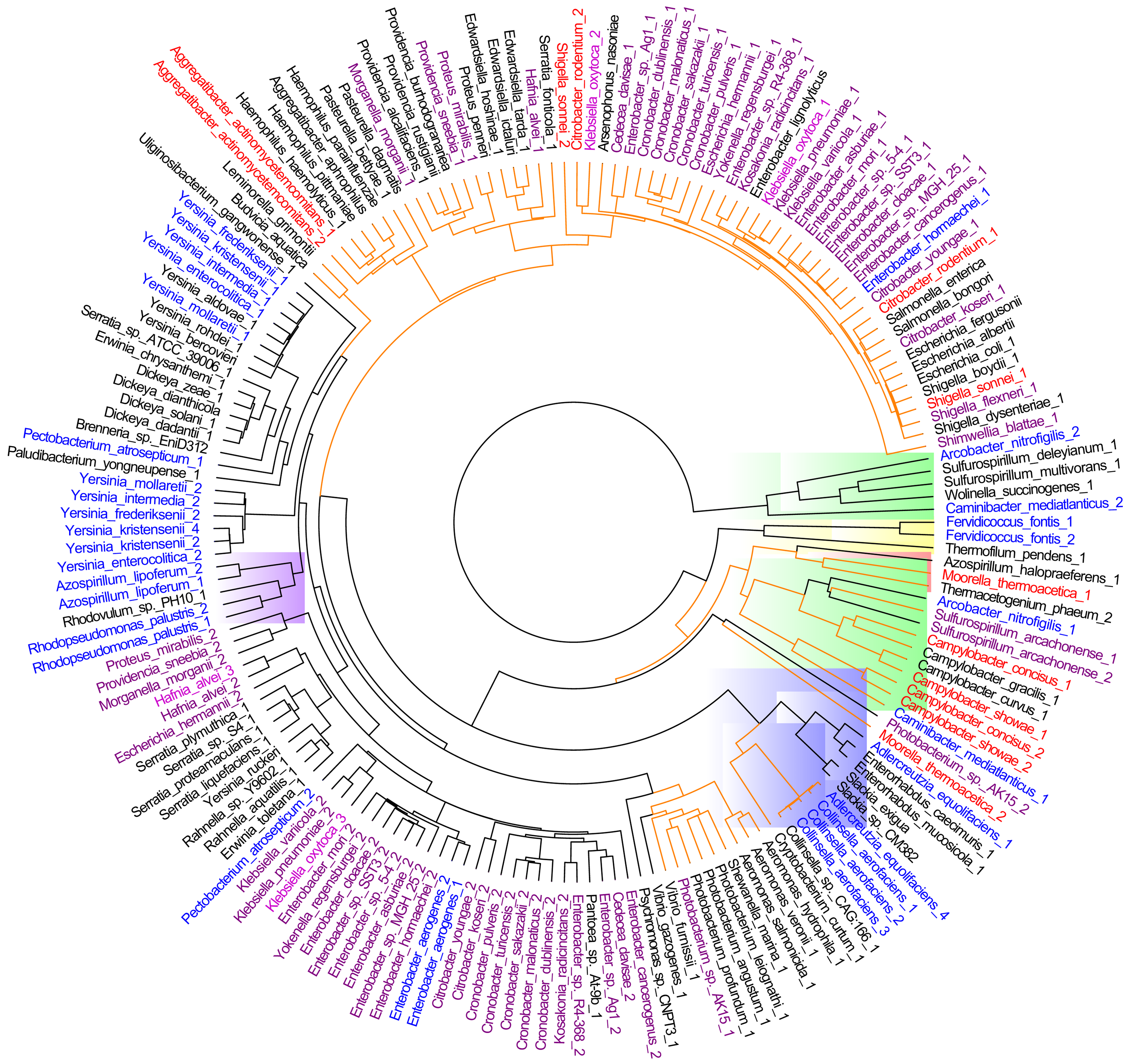


**Supp Figure S4: Phylogenetic analysis of metal-dependent formate dehydrogenases in prokaryotes also containing a Group 4A [NiFe]-hydrogenase catalytic subunit.**

A phylogenetic tree of FdhF-like formate dehydrogenase catalytic subunits. Proteins linked by the orange line are those predicted to contain an active site seleno-cysteine residue, while those linked by the black line are those predicted to contain an active site cysteine. The coloured sections represent different phylogenetic groups: brown section are Firmicutes; green section are the Epsilonproteobacteria; yellow section are the Crenarchaeota (Archaea); lilac section are Actinobacteria; purple section are Alpha-proteobacteria; and those with no highlight colour are the Gamma-proteobacteria. Species names highlighted in red text have genomes where two FdhF (seleno-Cys) can be identified; species names highlighted in purple text have genomes where two FdhF (one seleno-Cys, one Cys) can be identified; species names highlighted in pink text have genomes where more than two FdhF (mixture of seleno-Cys and Cys) can be identified; and species names highlighted in blue text have genomes where two FdhF (both Cys) can be identified. *P. atrospeticum* FdhF is listed here as ‘Pectobacterium_atrospeticum_1’, while ECA1507 is ‘Pectobacterium_atrospeticum_2’.


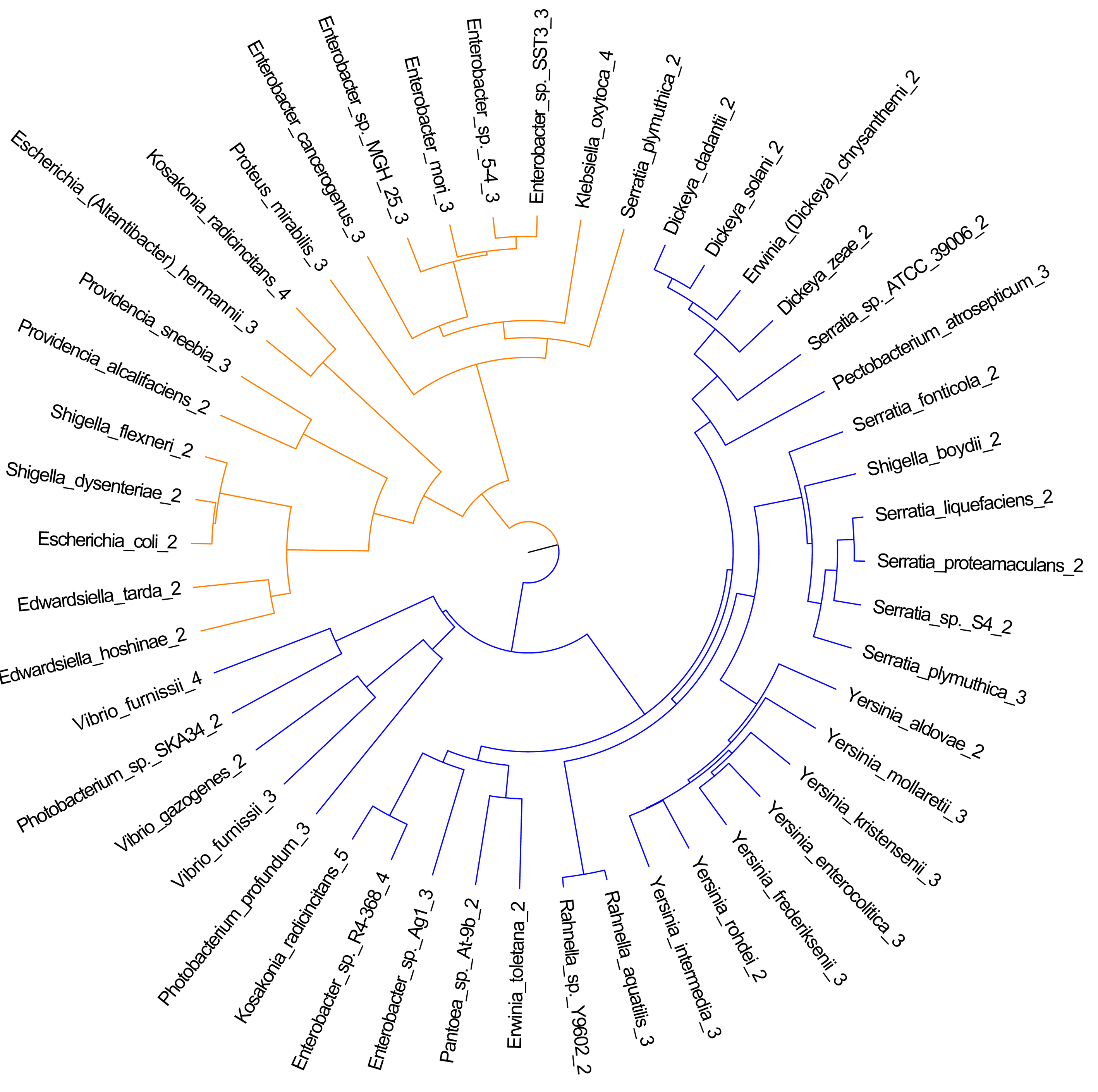


**Supp Figure S5: Phylogenetic analysis of YdeP proteins in prokaryotes.**

A phylogenetic tree of YdeP-like Mo/W-dependent catalytic subunits (similar to *P. atrospeticum* ECA1964 listed here as ‘Pectobacterium_atrospeticum_3’), which are related to formate dehydrogenases. Two separate clades are evident. YdeP is the protein product name used for the *E. coli* K-12 protein (listed here as ‘Escherichia_coli_2’).
